# Trophic structure of the macrofauna associated to deep-vents of the southern Gulf of California: Pescadero Basin and Pescadero Transform Fault

**DOI:** 10.1371/journal.pone.0224698

**Published:** 2019-11-05

**Authors:** Diana L. Salcedo, Luis A. Soto, Jennifer B. Paduan

**Affiliations:** 1 Posgrado en Ciencias del Mar y Limnología, Universidad Nacional Autónoma de México, Mexico City, Mexico; 2 Instituto de Ciencias del Mar y Limnología, Universidad Nacional Autónoma de México, Mexico City, Mexico; 3 Monterey Bay Aquarium Research Institute, Moss Landing, California, United States of America; Vrije Universiteit Brussel, UNITED STATES

## Abstract

Newly discovered hydrothermal systems in the Pescadero Basin (PB) and the neighboring Pescadero Transform Fault (PTF) at the mouth of the Gulf of California disclosed a diverse macrofauna assemblage. The trophic structure of both ecosystems was assessed using carbon (δ^13^C), nitrogen (δ^15^N), and sulfur (δ^34^S) stable isotopes. The δ^13^C ranged from -40.8 to -12.1‰, revealing diverse carbon sources and its assimilation via Calvin-Benson-Bassham and the reductive tricarboxylic acid cycles. The δ^15^N values were between -12.5 and 18.3‰, corresponding to primary and secondary consumers. The δ^34^S values fluctuated from -36.2 to 15.1‰, indicating the sulfide assimilation of biogenic, magmatic, and photosynthetic sources. In PB high-temperature vents, primary consumers including symbiont-bearing, bacterivores and filter-feeders predominated. The secondary consumers within the scavengers/detritivores and predator guilds were scarce. The siboglinid *Oasisia* aff *alvinae* dominated the macrofauna assemblage at PB, but rather than playing a trophic role, it provides a substrate to vent dwellers. In PTF low-temperature vents, only symbiont-bearing primary consumers were analyzed, displaying the lowest δ^34^S values. This assemblage was dominated by the coexisting siboglinids *Lamellibrachia barhami* and *Escarpia spicata*. δ^34^S values allowed to distinguish between PB and PTF vent communities, to exclude the presence of methanotrophic organisms, and the detection of photosynthetic organic matter input.

## Introduction

Ever since their discovery in 1977 in the Galapagos [1], the deep hydrothermal systems continue to disclose unique environmental conditions that influence the living and chemical conditions of the world oceans. These extreme environments are globally distributed along mid-ocean spreading centers and back-arc basins and host complex deep-sea ecosystems driven by sulfur-based chemosynthesis [1]. The hot hydrothermal fluids emerging from the seafloor are charged with sulfur reduced compounds, hydrocarbons and trace metals that contain the chemical energy necessary to support the metabolism of free-living and symbiont bacteria. Unlike photosynthesis based food-webs, these alternative systems sustain unusual trophic-webs where these bacteria constitute the primary producers.

So far, more than 720 active hydrothermal vent fields have been discovered around the world [2]. In 2012 and 2015, AUV mapping and ROV dives by the Monterey Bay Aquarium Institute (MBARI) in the southern Gulf of California (GoC) discovered hydrothermal vent sites on the Alarcón Rise (AR), the northern-most spreading segment of the East Pacific Rise (EPR), on the Tamayo and Pescadero Transform Faults that bound the AR [3], and in the nearly 3,800 deep, sediment-filled Pescadero Basin (PB) [4]. The hydrothermal vent communities in PB, the adjacent Pescadero Transform Fault (PTF), and on the AR are distinct from each other [5], despite their proximity. This fact raises new questions concerning the role of habitat and fluid chemistry, determining the metabolic pathways available to the organisms and carbon flow through the system.

A trophic-web depicts the exchange of organic matter (OM) among organisms and the energy flow from basal resources to top predators [6]. A vent trophic-web is commonly structured into the following groups: chemosynthetic primary producers and other microbes at the base of the food web; primary consumers; secondary consumers, and parasites [7,8]. Stable isotope analysis (SIA) has become a widespread and versatile tool to unravel trophic-webs structure and functioning, as it encompasses biological and environmental information. SIA employs the small natural variations in stable isotope ratios resulting from physical, chemical, and biological processes that cause isotope fractionation [9]. Most of the trophic-web studies are based on a dual isotope approach, using δ^13^C and δ^15^N. However, the complementary use of δ^34^S values has recently been taking relevance and has added evidence on the possible role of the symbiont-containing animals in the nutrition of the heterotrophic fauna. δ^13^C provides information on the carbon/energy sources at the base of the food-web [10,11,12]; in hydrothermal systems, δ^13^C values of the symbiont-bearing organisms also reflect the carbon fixation pathway of their symbionts [13]. The main carbon fixation pathways in hydrothermal vent food-webs are the Calvin-Benson-Basham (CBB) (δ^13^C < 22‰) and the reductive tricarboxylic acid (rTCA) (δ^13^C > -16‰) cycles [13,14]. δ^15^N is used to estimate the trophic position of a consumer [15], as its values are typically enriched by 2–4‰ relative to their diet [16]. δ^34^S in the fauna is useful in distinguishing energy sources [14,17].

It is hypothesized that the structure and ecological functioning of the trophic-web of two deep-sea systems, high-temperature vents in Pescadero Basin and low-temperature vents in the Pescadero Transform Fault, may differ due to the inherit habitat complexity of the systems. Food-web dissimilarity among active sites may involve different fluid regimes, substrate heterogeneity, and distinct physical-chemical conditions. Therefore, the objective of this study was to describe the trophic structure of the macrofauna of these chemosynthetic ecosystems, and to conduct a comparative analysis of them, through the employment of stable isotopes as biomarkers. The use of δ^13^C, δ^15^N, and δ^34^S allows the identification of the potential sources supporting the food-web of the vent and non-vent macrofauna assemblage. It is also expected to elucidate trophic levels and diversity guilds.

## Materials and methods

### Study area

**Fig 1:**
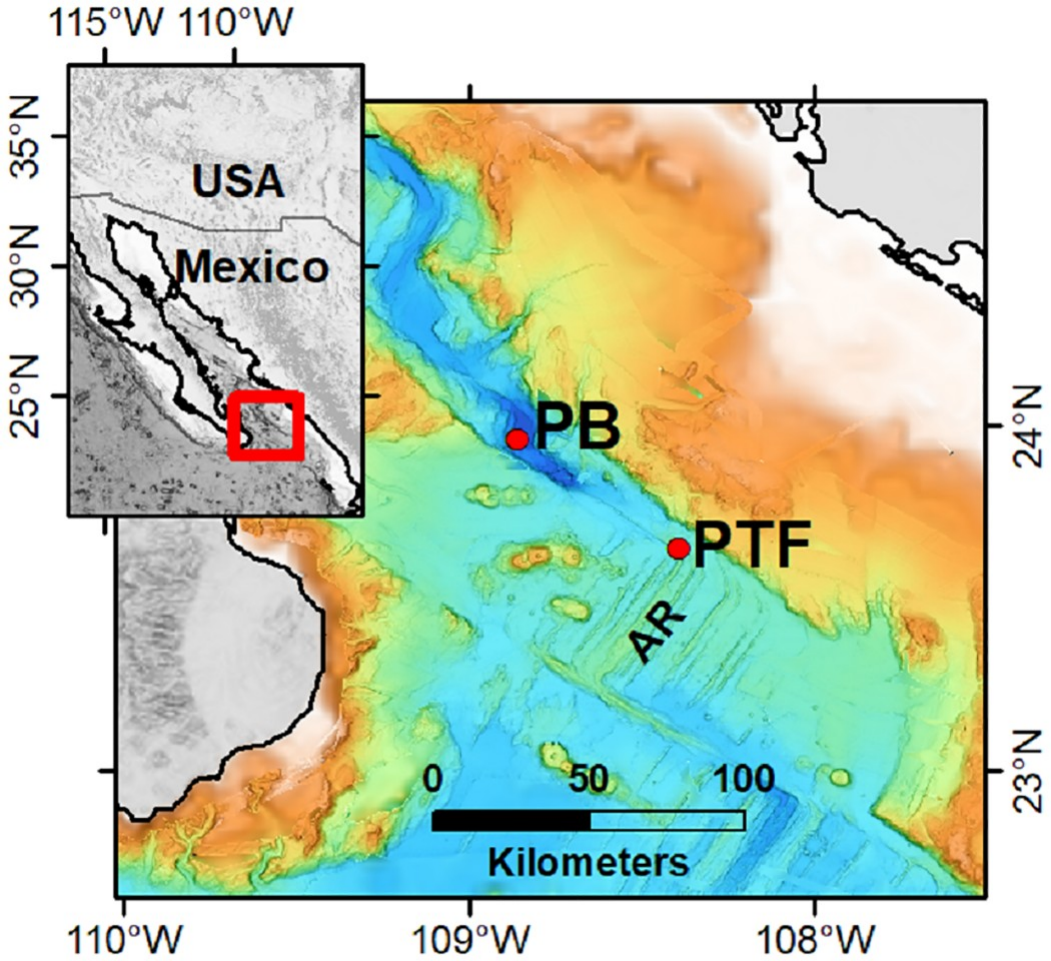
Location of the study area in the southern Gulf of California, Mexico. Vent sites are red dots. PB = Pescadero Basin. PTF = Pescadero Transform Fault.

The Gulf of California is a semi-enclosed and oceanographically dynamic basin located at 22°-32° N, and 105°-107° W in the northeastern Pacific Ocean (Fig 1). The mouth of the gulf, located in the Eastern Tropical Pacific off the Mexican coast, is a transitional region [18,19].

The Pescadero Basin is a deep sedimented rift graben near the entrance of the GoC, at 24° N (Fig 1). The PB vent field named Auka extends over an area of approximately 0.2 by 0.5 km along a fault scarp on the southwestern edge of the basin at about 3,670 m depth. It includes a series of carbonate mounds and chimney edifices [4] containing calcite and anhydrite with sulfide, and harboring dense colonies of the siboglinid tubeworm *Oasisia* aff. *alvinae* [5]. Vent fluids reach up to 291°C and contain high concentrations of dissolved gases, including higher-order hydrocarbons, methane (CH_4_), CO_2_, and hydrogen sulfide, while helium concentration is low [4.5]. The fluids have elevated chlorinity at 646 nmol/kg, a pH of 6.3 an d are enriched in highly soluble elements such as B and alkalis, with high Li, Rb, and Cs contents [4]. Trace metals such as Fe, Mn, Cu, and Zn are also present, their concentrations can be consulted in Paduan *et al*. [4]. The PB vents host a faunistic assemblage composed of 27 taxa identified so far, of which 17 are unknown at other regional vents, and many are new species [5].

The Pescadero Transform Fault extends for 60 km between the Pescadero Basin and Alarcón Rise. The known vent sites are at SE at 23.64° N and 2,400 m depth (Fig 1). They are low-temperature hydrothermal vents located at the periphery of a hill of thick sediment that was uplifted by a volcanic sill [3], from which lava flows erupted. Fluids emanating from the vents had a temperature of 5°C [3], but no fluid samples were collected. In this site, 15 taxa were identified, and it is dominated by dense clusters of coexisting siboglinid tubeworms *Escarpia spicata* and *Lamellibrachia barhami*. The endosymbiont-bearing vesicomyids *Calyptogena* and *Archivesica* occupy surrounding soft-sediments [5].

### Biological sampling and stable isotopes analysis

The Pescadero Transform Fault was mapped at high resolution in 2012 on an expedition with the R/V *Zephyr* [3], and the Pescadero Basin in 2015 on an expedition with the R/V *Rachel Carson* [4], using the MBARI autonomous underwater vehicle (AUV) *D*. *Allan B*. The PB and PTF were explored and sampled in 2015 on dives with the remote operated vehicle (ROV) *Doc Ricketts*, during which the vents on the PTF were discovered [3,4,5]. The sampling was carried out in compliance with the necessary permits granted by the compelling Mexican authorities.

Biological samples were collected at the PB and PTF vent sites in 2015 by MBARI with the ROV *Doc Rickett*s. The sampling areas included the high-temperature flows at chimneys and mounds in the PB and the low-temperature vents of PTF. The sampling effort was unbalanced between localities since three dives were conducted in PB, while only one was carried out in PTF. The vent-fauna was sampled from active sites (both chimneys and diffuse flow sites), including siboglinid colonies and clam-beds, aiming at the most conspicuous taxa with different trophic strategies. The non-vent fauna was collected in the surrounding sediments or basalts, where no apparent signs of hydrothermal influence were observed. The organisms were identified to the lowest possible taxonomic level and stored frozen at -80°C.

For isotopic analyzes of carbon (δ^13^C), nitrogen (δ^15^N), and sulfur (δ^34^S), the animal tissues were dissected into individual organs whenever possible. Assuming potential differences in carbon isotopic ratios in soft tissues hosting bacteria in symbiont-bearing organisms, vesicomyid clams were dissected into the gills, foot, and mantle, whereas large siboglinid worms were separated into gill plumes, vestimentum, and trophosome. Muscle tissue samples were dissected from larger crustaceans. Due to their size or complex anatomy, small to medium-size organisms were used whole after removal of the digestive tract. Small individuals were pooled to obtain a minimum sample of 2 mg of dry weight. Tissues were thawed, washed with distilled water, dried at 60°C, ground and homogenized using an agate mortar and pestle. The samples were analyzed on a Thermo Finnigan Delta Plus XL connected to a Carlo Erba EA (model 1005) via Conflo III. The isotopic determinations were made at the Stable Isotope Ratio Facility for Environmental Research (SIRFER), University of Utah. Values of δ^13^C are reported relative to Vienna Peedee Belemnite (VPDB) scale, δ^15^N values relative to the AIR scale, and δ^34^S relative to Vienna-Canyon Diablo Troilite (VCDT). Delta notation as parts per thousand (‰) was used. Analytical errors associated with the overall process of these determinations were between 0.1 and 0.3‰.

Biplots to analyze the relationships among nitrogen and sulfur isotopic ratios *vs*. carbon isotopic ratios were constructed. When possible, species were classified in trophic guilds after consulting the literature. Species with unknown or uncertain diets were included in trophic guilds considering feeding strategies of close related species and complementary using the isotopic values herein obtained. A trophic diagram was constructed following Tunniclife´s [7].

## Results

### Stable isotopes values

**Fig 2:**
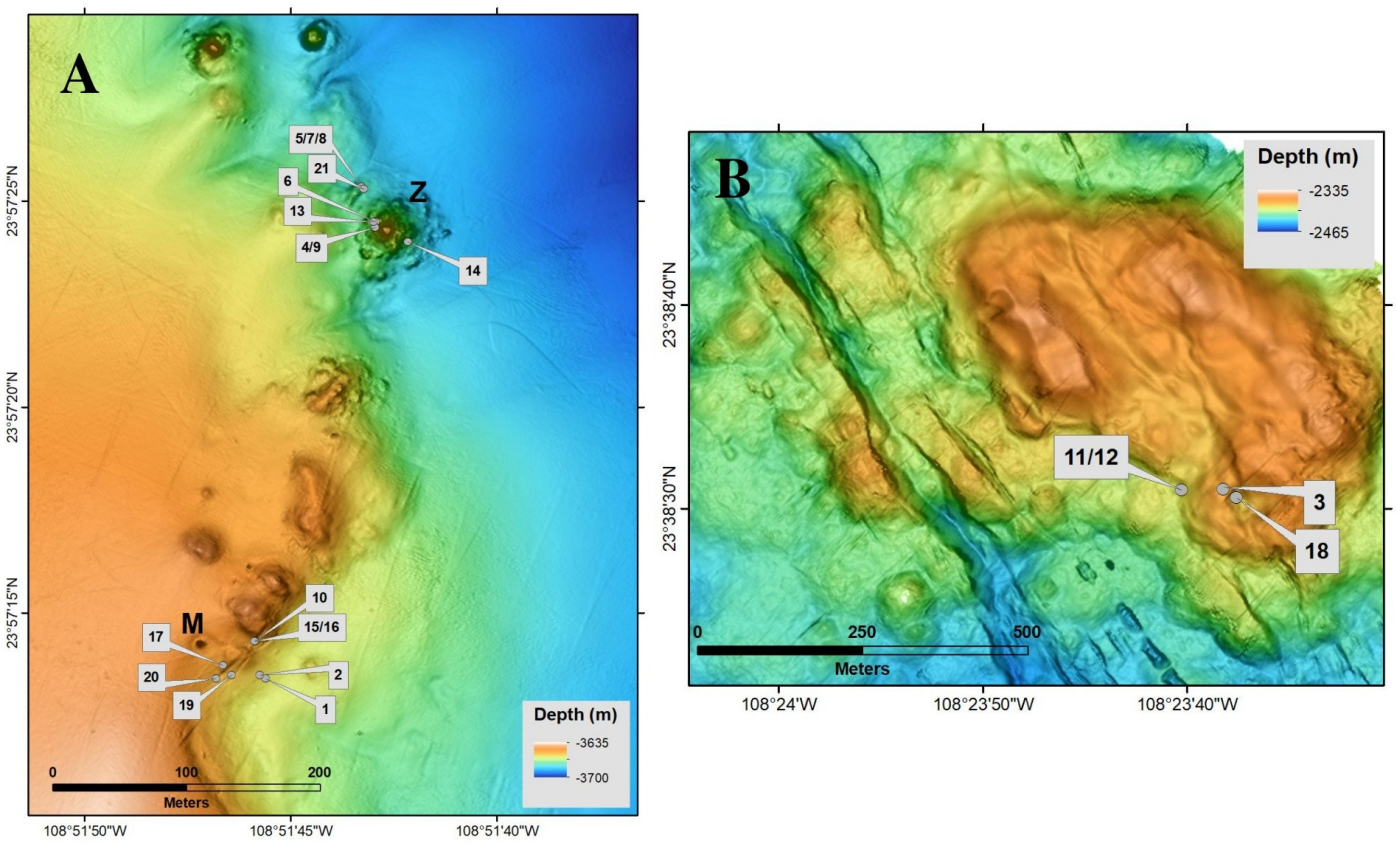
Spatial distribution of the biological samples collected. A) Pescadero Basin (PB) and B) Pescadero Transform Fault (PTF). Numbers identifying individual points are listed in Table 1 as ID numbers.

A total of 1,283 animal specimens belonging to 21 species was analyzed, 17 from Pescadero Basin (Fig 2A) and 4 from Pescadero Transform Fault (Fig 2B). Data from both localities were analyzed together. The δ^13^C, δ^15^N, and δ^34^S values of the specimens collected are summarized in Table 1.

**Table 1:** δ^13^C, δ^15^N, and δ^34^S individual or average values of the vent and non-vent fauna from Pescadero Basin (PB) and Pescadero Transform Fault (PTF).

TAXON	ID	TISSUE	TROPHIC GUILD	N	δ^13^C‰	δ^15^N‰	δ^34^S‰
**CNIDARIA**							
Actiniaria sp. 1 (PB)	1.1	oral disk	symbiont-bearing	1	-38,2	3,0	N/A
	1.2	columella		1	-35,6	2,7	7,1
Actiniaria sp. 2 (PB)	2.1	oral disk	symbiont-bearing	1	-34,5	6,3	3,7
	2.2	columella		1	-32,6	6,8	3,9
Actiniaria sp. 3 (PTF)	3.1	oral disk	filter-feeder	1	-19,4	**18,3**	14,9
	3.2	columella		1	-17,8	18,3	**15,1**
Actiniaria sp. 4 (PB)	4	complete specimen	filter-feeder	6**	-23,6	4,5	-10,4
Actiniaria sp. 5 (PB)	5.1	oral disk	symbiont-bearing	1	-38,1	0,7	-0,5
	5.2	columella		1	-37,9	1,0	1,7
Zoantharia sp. (PB)	6	complete specimens	filter-feeder	26**	-20,8	14,0	9,8
**ANNELIDA**							
Amphinomidae sp. 1 (PB)	7	complete specimen	scavenger/detritivore	1	-17,9	6,7	-13,6
Amphinomidae sp. 2 (PB)	8	complete specimen	scavenge/detritivore	1	-22,3	4,5	-2,6
*Oasisia* aff. *alvinae* (PB)	9	complete specimens	symbiont-bearing	6	-14,6 ± 1,33	1,97 ± 0,37	-14,55 ± 1,73
*Riftia pachyptila* (PB)	10.1	gill plume	symbiont-bearing	1	-13,2	3,6	-10,5
	10.2	vestimentum		1	-13,1	3,8	-10,6
	10.3	trophosome		1	**-12,1**	3,0	-7,8
	10.4	opisthosome		1	-13,3	3,7	-10,5
*Lamellibrachia barhami* (PTF)	11.1	vestimentum	symbiont-bearing	2	-16,95 ± 1,05	-1,15 ± 1,46	-35,07 ± 0,66
	11.2	trophosome		2	-15,68 ± 3,67	-1,99 ± 1,62	-32,96 ± 1,02
*Escarpia spicata* (PTF)	12.1	vestimentum	symbiont-bearing	1	-17,4	0,5	**-36,2**
	12.2	trophosome		2	-20,13 ± 3,01	-0,28 ± 0,81	-32,44 ± 2,04
*Paralvinella* sp (PB)	13	complete specimens	bacterivore	11	-19,52 ± 1,39	-0,55 ± 0,28	-8,61 ± 3,09
*Ophryotrocha* cf. *akessoni* (PB)	14	complete specimens	bacterivore	1100**	-25,1	-0,3	4,6
Polynoidae sp. (PB)	15	complete specimens	scavenger/detritivore	2**	-13,6	5,4	-7,6
*Nereis* cf. *sandersi* (PB)	16	complete specimen	scavenger/detritivore	1	-35,8	4,6	4,4
**MOLLUSCA**							
*Archivesica* sp. 7 (PB)	17.1	foot	symbiont-bearing	2	-34,8 ± 0,8	-2,5 ± 0,3	-7,9 ± 3,2
	17.2	mantle		1	-35,6	-2,3	-0.9
	17.3	gill		2	-34,8 ± 1,69	-3,9 ± 1,62	-9,7 ± 2,01
*Calyptogena costaricana* (PTF)	18.1	foot	symbiont-bearing	1	-39,5	-9,1	-26,4
	18.2	mantle		1	-36,4	-8,8	-26,8
	18.3	gill		1	**-40,8**	**-12,5**	-32,7
*Provanna laevis* (PB)	19	complete specimens	bacterivore	100**	-27,0	4,3	7,6
**CRUSTACEA**							
*Munidopsis scotti* (PB)	20	muscle	predator	1	-19,2	10,5	0,8
**PYCNOGONIDA**							
Pycnogonida sp. (PB)	21	complete specimen	scavenger/detritivore	1	-26,8	6,0	-0,6

**Pooled organisms.

The δ^13^C values of the fauna ranged from -40.8 to -12.1‰. The lowest ratios corresponded to the vesicomyid clam *Calyptogena costaricana* (Table 1), found in PTF (Fig 2B). In contrast, the highest values corresponded to the siboglinid worms *O*. aff. *alvinae* and *Riftia pachyptila* (Table 1), observed in PB vents (Fig 2A). The δ^15^N values were between -12.5 and 18.3‰. The lowest ratios were also recorded in the vesicomyid clam *C*. *costaricana* (Table 1), whereas the highest corresponded to the unknown species Actiniaria sp. 3 (Table 1). This anemone is an epicommensal of a pagurid crab and was captured in the surrounding sediments off PTF vents (Fig 2B). The δ^34^S values varied between -36.2 and 15.1‰. The lowest ratios corresponded to the siboglinid tubeworms *L*. *barhami* and *E*. *spicata* from the PTF (Table 1). The highest value was observed in Actiniaria sp. 3 (Table 1).

**Fig 3:**
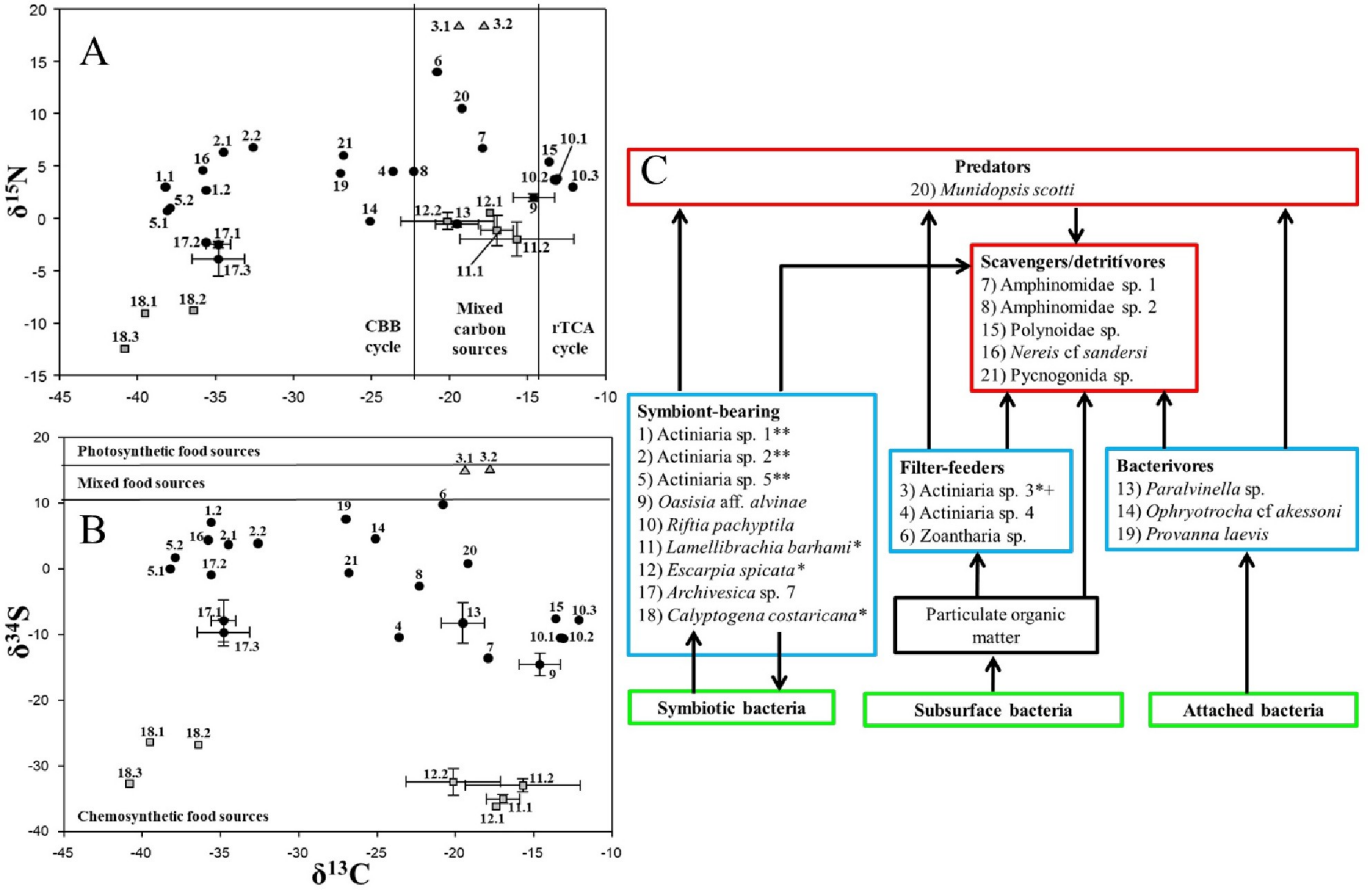
Dual isotope plots showing stable isotope signatures (‰) of macrofaunal species collected in PB and PTF. A) δ^13^C *vs*. δ^15^N. B) δ^13^C *vs*. δ^34^S. ● Fauna from PB. ■ Fauna from PTF. ▲ Non-vent fauna. C) Trophic diagram of the macrofauna of PB and PTF *Species from PTF. **Species whose feeding guild is not confirmed. + Non-vent fauna. Numbers identifying individual points are listed in Table 1 as ID numbers.

The wide range of δ^13^C values indicated the existence of diverse carbon sources. Our results revealed that the studied fauna employ two metabolic pathways to fix organic carbon: the Calvin-Benson-Bassham cycle (CBB), and the reductive tricarboxylic acid cycle (rTCA). The δ^13^C *vs*. δ^15^N diagram showed similar patterns detected in other hydrothermal systems: symbiont-bearing species displayed the lowest ratios of both isotopes, while the top predator *M*. *scotti* had the highest δ^15^N value, only surpassed by the Zoantharia sp. and the non-vent Actiniaria sp. 3 (Fig 3A). Nearly 50% of the species analyzed relied on organic carbon fixed through the CBB, while only two species (9%) assimilated carbon derived from rTCA (Fig 3A). The remaining organisms consumed mixed carbon sources (Fig 3A). The δ^13^C *vs*. δ^34^S biplot showed that most of the organisms consumed sulfur of chemosynthetic origin, except Actiniaria sp. 3. The lowest δ^34^S values corresponded to PTF inhabitants (Fig 3B). In both biplots, the non-vent species was depicted at the top, with both high δ^15^N and δ^34^S values (Fig 3B).

### Trophic structure

Among the Pescadero Transform Fault species analyzed, only primary consumers were identified, all of them in symbiosis with thiotrophic bacteria: *C*. *costaricana*, *E*. *spicata*, and *L*. *barhami* (Fig 3C). In the periphery of PTF, only Actiniaria 3, a non-vent organism, was analyzed.

In Pescadero Basin vents, a much complex trophic-web was recognized with two trophic levels: primary consumers and secondary consumers. Among the primary consumers, three trophic guilds were determined, 1) symbiont-bearings (δ^13^C = -40.8 to -12.1‰; δ^15^N = -12.5 to 6.8‰, δ^34^S = -36.2 to 7.1‰, individual values), 2) filter-feeders (δ^13^C = -23.6 to -20.8‰, δ^15^N = 4.5 to 14‰, δ^34^S = -10.4 to 9.8‰, individual values), and 3) bacterivores (δ^13^C = -27 to -18.5‰, δ^15^N = -0.8 to 4.3‰, δ^34^S = -6.4 to 7.6‰, individual values). The first guild encompassed 9 species (Fig 3C) and was dominant both in biomass and species richness. Actiniaria sp. 1, sp. 2, and sp. 5 were assigned to this group considering their isotopic ratios, but further molecular analyzes should be done to confirm this assumption. The second guild was integrated by two species, Actiniaria sp. 4 and Zoantharia sp. (Fig 3C), although the last species showed an anomalously high δ^15^N value. The third guild included polychaete worms and a mollusk (Fig 3C). The secondary consumers included two groups, 1) scavengers/detritivores (δ^13^C = -35.8 to -13.6‰, δ^15^N = 4.5 to 6.7‰, δ^34^S = -13.6 to 4.4‰, individual values), and 2) predators (δ^13^C = -19.2‰, δ^15^N = 10.5‰, δ^34^S = 0.8‰). The first guild included mostly polychaete worms and the Pycnogonida sp., while the second only the galatheid *M*. *scotti*, in low densities (Fig 3C).

The symbiont-bearings had the widest intervals of all the analyzed isotopes, and their ratios overlapped to those of organisms belonging to other trophic guilds. However, the other trophic groups were distinguished by their δ^15^N ranges: bacterivores (-0.8 to 4.3‰), scavengers/detritivores (4.5 to 6.7‰), and predators (10.5‰). The filter-feeder guild had a δ^15^N range of 4.5 to 14‰ that overlapped with the ratios of the secondary consumers; however, this was due to the anomalously high δ^15^N value of Zoantharia sp.

## Discussion

### Carbon isotope ratios (δ^13^C) and fixation pathways

Sulfide (S^2-^) oxidation is the primary energy source that vent microorganisms use for carbon fixation. The main carbon fixation pathways in the hydrothermal vent food-web are the CBB and the rTCA cycles [14,20]. Methane oxidation (methanotrophy) is another carbon fixation process taking place at vents and seeps. The vertical flux of both dissolved organic carbon (DOC) and particulate organic carbon (POC) of autotrophic origin may also provide some nutrition to vent macroconsumers [14]. The intervals of δ^13^C values observed in the fauna of PB and PTF are comparable to others detected in similar ecosystems, in Juan de Fuca Ridge [21], Galapagos Rift [22], and Guaymas Basin [23].

The vesicomyid *C*. *costaricana* from PTF showed the lowest δ^13^C (-40.8 to -36.4‰), particularly in its gill tissues, where the endosymbionts are hosted (Table 1). These ratios approach those of methane (CH_4_) reported in Guaymas Basin sediments (-79 to -37.9‰) [24,25]. Presumably, this clam could use CH_4_ as a carbon source [5]. However, no methanotrophic endosymbionts have been found in its tissues. Despite of the availability of methane as a potential carbon source in PTF vents, the animals of this locality do not use it as an energy source. Methane is likely to be consumed only by bacterial consortiums, which may produce depleted carbon further assimilated by metazoans.

The vesicomyid *Archivesica* sp. 7 from PB also presented low δ^13^C values ranging from -35.6 to -34.8‰, with the lowest recorded in the gills. These depleted ratios are shared among clams with thiotrophic endosymbionts [26]. Thus it is expected that this primary consumer obtains its energy through the sulfide oxidation performed by its endosymbionts, and that fixes carbon through the CBB cycle.

The anemones are commonly recognized as filter-feeders. However, the isotopic analyzes performed in Actiniaria sp. 1, sp. 2 and sp. 5 showed unexpected low δ^13^C values (-38.1‰ to -32.6) close to those of the vesicomyids. These ratios differ from those commonly recorded in filter-feeders. The trophic guild of these actiniarids is uncertain, but based on these findings, they were considered as symbiont-bearings. An anemone from Gorda Ridge had a similar δ^13^C value (-31.3‰), but it was attributed to the assimilation of an indistinct depleted carbon source [21]. A symbiotic relationship between vent anemones and thiotrophic bacteria is currently unknown, but this trophic strategy might be compatible with such depleted values. For instance, bacterial consortiums have been identified in the tentacles of the shallow-water anemone *Metridium senile* [27].

Interestingly, the lowest carbon ratios of the actiniarids herein analyzed were recorded in the oral disk, in comparison to the columella, similarly to the detected pattern in vesicomyids gills (Table 1). Furthermore, sulfur-oxidizing endosymbiotic bacteria such as *Sulfurimonas*, *Helicobacter*, *Sulforovum*, and *Sulfospirillum* have been studied in the intertidal anemone *Anemonia viridis*. These bacteria have also been found in the hydrothermal systems off of Okinawa and deep vents off the northeast Pacific Ocean [28]. It is possible that these anemones have developed both feeding strategies, similarly to mussels and clams, given their direct exposure to diffuse fluids.

The giant worm *R*. *pachyptila* displayed the highest δ^13^C values (Table 1, Fig 3A), similar to those reported in the Guaymas Basin [23]. Individuals with values >-16‰ [14] tend to assimilate the carbon through the rTCA cycle, assuming a net fractionation between -2 and -14‰ among the inorganic substrate and the organic product [14]. Interestingly, Candidatus *Endoriftia persephone*, the endosymbiont bacteria of *R*. *pachyptila*, is the first identified bacterium able to express both CBB and rTCA carbon fixation pathways simultaneously [29]. Previous records indicate that hyperthermophiles macro-consumers employ the rTCA pathway in the assimilation of organic carbon. This cycle is the dominant pathway in habitats between 20°C and 90°C [13]; hence, it is assumed that this siboglinid is exposed to fluids with temperatures above 20°C in PB.

*O*. aff *alvinae* showed individual high carbon isotopic ratios (-16 to -12.2‰) that place it closer to the values corresponding to the carbon fixation through the rTCA cycle. This siboglinid worm is a foundation species forming three-dimensional biogenic structures that increase the potential number of ecological niches, providing a substrate for colonization by other species, and offers a refuge against predation and extreme abiotic conditions [30]. This siboglinid worm presented high densities of up to 2,400 individuals/m^-2^ in PB [5], but the low degree of exploitation of its biomass and a potential source of food suggests that rather than playing a trophic role, *O*. aff. *alvinae* primarily plays a structural role in this vent system.

As in other vent communities [31], the worms *R*. *pachyptila* and *O*. aff *alvinae* exhibited higher δ^13^C values compared to vesicomyids. The worm and clam symbionts employ different pathways to fix carbon, resulting in different fractionation proportions. In PTF, the siboglinid *E*. *spicata* and *L*. *barhami* also had higher δ^13^C values than *C*. *costaricana*, showing that these worms consume mixed carbon sources, derived from both CBB and rTCA cycles, while vesicomyids utilize the CBB cycle. The CBB cycle appears to be the main pathway in habitats with temperatures <20°C [13]. This finding is consistent with the exposure of both vesicomyid species to diffuse fluids emerging from the sediments, which might have lower temperatures than the fluids emerging directly from the chimneys.

The Polynoidae sp. was detected among siboglinid tubes, but this scavenger/detritivore does not feed on *R*. *pachyptila*, since it had a lower δ^13^C. However, this polynoid presented a δ^13^C value around 1‰ enriched with respect to *O*. aff *alvinae*, indicating a potential predatory activity on tubeworms, as it has been observed in Juan de Fuca Ridge vents [30].

The dorvilleid polychaete *O*. cf. *akessoni* was observed in high abundances crawling on the tubes of *O*. aff. *alvinae*, sometimes smothering them completely. However, its δ^13^C signature was lower than those of the siboglinids, thus suggesting that this species does not feed on them. Dorvilleids use the tubeworms as a substrate and probably feed on free-living CBB bacteria and detritus attached to the tubes, which place this species as a primary consumer with a bacterivore feeding strategy. Space and nutritional resources monopolization by this worm may reduce the settlement of other vent species, even by potentially grazing recruits [30]. Vent macro-consumers with δ^13^C values lower than -22‰ assimilate the fixed carbon through the CBB cycle because the total fractionation associated with inorganic carbon fixation to organic by the RuBisCO I form ranges from -22 to -30‰ [14]. Including both PB and PTF, the organisms with values lower than -22‰ encompassed the polychaetes Amphinomidae sp.2, *N*. cf *sandersi*, and *O*. cf *akessoni*, and the Pycnogonida sp., all within the scavenger/detritivore guild, as well as the filter-feeder Actiniaria sp. 4 and the bacterivore gastropod *Provanna laevis*. It is assumed that these species, and the bacterial consortiums from which they feed are exposed to temperatures below 20°C, where the CBB cycle works properly [13].

The δ^13^C signatures ranging from -16 to -22‰ indicate a mixture of carbon sources assimilated by the fauna [14]. The polychaetes *Paralvinella* sp., and Amphinomidae sp. 1, the Zoantharia sp, and the crustacean *M*. *scotti* fall within that interval. These organisms may be consuming free-living bacteria, or they are detritivores or scavengers that use different trophic routes. In the case of the bacterivores, we inferred that they feed on a heterogeneous mixture of bacteria consortiums that fix carbon through both CBB and rTCA pathways. The wide range of individual δ^13^C signatures in bacterivores (-27 to -18.5‰), coupled with the absence of isotopic niche overlapping, suggest a heterogenic free-living bacterial pool with different isotopic ratios, which may be linked to the diversity of microhabitats and environmental conditions of PB vents. The non-vent Actiniaria sp. 3 had an overlapping carbon isotopic ratio within the same interval, corresponding to the assimilation of a mixture of carbon sources, which makes δ^13^C useless to separate it from vent-fauna.

The low δ^13^C recorded for the provanid gastropod *P*. *laevis* suggested a dependence on a specific free-living microbial community that relies on a depleted carbon source. So far, the presence of endosymbionts in this gastropod has not been reported, but similar symbiotic relationships have been described in other provannid species [30,32].

### Nitrogen isotope ratios (δ^15^N), sources and trophic guilds

The ample trophic discrimination in δ^15^N values (from 2 to 5‰) between the consumer and the diet provides information about the trophic position of an organism in relation to a primary consumer [33]. However, vent organisms have δ^15^N values that are associated with the nitrogen origin or to local biogeochemical processes [34]. Previous reported δ^15^N values for soft tissues from thiotrophic and methanotrophic animals range from -20 to +7‰ [26,35].

As in the case of δ^13^C ratios, the lowest δ^15^N values corresponded to the vesicomyid clams *C*. *costaricana* from PFT and *Archivesica* sp. 7 from PB. Such low values probably reflect the effect of isotopic fractionation by the enzymes of the endosymbionts lodged in their gills [35,36]. Low values were also observed in the tubeworms *E*. *spicata* and *L*. *barhami*. Such depleted δ^15^N values in marine animals have been reported only in communities consisting of chemosynthesis-based animals and cyanobacteria [13]. Organisms bearing chemosynthetic bacteria generally have depleted values ranging from -13 to 5‰, compared to values of 5 to 15‰ observed in other marine organisms [37]. This condition has also been observed in the Galapagos Rift hydrothermal vents (1.8 to 9.8‰) [38] and Juan de Fuca, from -10‰ to 4‰ [39], and from -8.5 to 9.4‰ [30].

*R*. *pachyptila* had values among 3 and 3.8‰, similar to those previously recorded for the same species in Genesis, Parigo, and Elsa sites on the EPR (from -2.9 to 5‰) [37]. The oxidizing bacteria that live in symbiosis with this siboglinid worm exclusively assimilate NO_3_, unlike most vent-fauna that use NH_4_ [37]. In the process of assimilation of NO_3_ and NH_4_, and the production of biomass by bacterial consortiums, a high isotopic fractionation occurs. This is another factor causing depleted δ^15^N values in vent fauna [38]. *O*. aff. *alvinae* had nitrogen isotopic ratios (1.5 to 2.4‰) close to those of *R*. *pachyptila*. The tubeworms *E*. *spicata* and *L*. *barhami* coexist in dense colonies in the vents of PTF; given their proximity, one would expect that they share a mutual nitrogen source. Furthermore, analyzes of 16S ribosomal RNA sequences indicate that these siboglinids house the same endosymbiont species. However, they differ in their δ^15^N ratios, implying that they are either using a different nitrogen chemical species or are discriminating differently after the nitrogen uptake.

The worms *Paralvinella* sp. and *O*. cf *akessoni* and the gastropod *P*. *laevis* presented low δ^15^N values close to 0‰ (Table 1) and were here considered as bacterivores. Their isotopic ratios may be related to nutrition based on microbial consortiums that depend on local nitrogen sources. Both species occupy different spatial and isotopic niches (Fig 2A), *Paralvinella* sp. inhabits crevices directly exposed to diffuse fluids, while *O*. cf *akessoni* crawls on *O*. aff *alvinae* tubes, which are also in close contact with the emanations, so they presumably feed on different microbial consortiums that depend directly from the discharged sulfides [37].

The Pycnogonida sp., both species of Amphinomidae, the Polynoidae sp. and *N*. cf *sandersi* presented intermediate δ^15^N values proper of secondary consumers included in the scavengers/detritivore guild. These species occupy different spatial niches, and they probably feed on smaller invertebrate preys not analyzed in this study. The Polynoidae sp. showed a trophic enrichment of about 3.5‰ respect to *O*. aff *alvinae*. Therefore, it could be a food item together with other small prey as it was inferred from the δ^13^C analysis.

The top consumer within the PB vents was the galatheid crab *M*. *scotti*, with a high δ^15^N value of 10.5‰. It was found near *R*. *pachyptila* tubes and presented a trophic enrichment superior to 7‰ with respect to this worm. However, considering its low δ^13^C value, it does not seem to prey on this siboglinid. This galatheid is not a specialized predator since it includes several preys in its diet, potentially including amphinomid worms.

The highest δ^15^N values corresponded to the non-vent Actiniaria sp. 3 (18.3‰). Actiniarids from the periphery of the vent fields share positive δ^15^N values, although they seem to occasionally profit from the vent community byproducts [40]. Non-vent deep-sea fauna usually has higher δ^15^N values than vent-fauna [22,23,36,41]. Values between 14.5‰ and 17.9‰ were recorded in non-vent organisms collected near the vents of the Guaymas Basin [23]. The high δ^15^N values of the episymbiont Actiniaria sp. 3 corresponded to the assimilation of a mixture of OM both of photosynthetic and chemosynthetic origin. The other Actiniaria species had relatively high δ^15^N values. Although sea-anemones are usually considered as peripheral species at hydrothermal vents occupying the zone of oxidized sulfide [42], in this study, they were abundant on chimney walls and sometimes they were directly exposed to the shimmering fluids. A species of zoanthid observed in scattered carbonates and sediments with emerging fluids [5] presented a δ^15^N value of 14‰, anomalously high for a primary vent consumer. This zoanthid is a filter feeder, possibly using various nitrogen sources (NH_4_, DIN, and NO_3_).

### Sulfur isotope ratios (δ^34^S) and sulfur sources

The origin of sulfur in hydrothermal systems is twofold: magmatic sulfur and sulfate from seawater, and its isotopic values in these systems are determined by the mixing ratio of both components. The δ^34^S values of basaltic sulfur are usually close to 0‰, and close to 5‰ in rhyolitic sulfur. On the other hand, major sulfur species in hydrothermal solutions are sulfate and hydrogen sulfide. The light isotope of sulfur is favored in H_2_S (S^2-^) compared to sulfates (SO_4_^2-^). Thus, magmatic H_2_S are significantly depleted compared to their source of magmatic sulfur due to fractionation processes [43].

The δ^34^S ratios of vent-fauna closely reflect the isotopic values of reduced sulfur discharged through the vents, and therefore, can be used to identify dominant sources of sulfur (magmatic or biogenic) [43], because there is little or no fractionation during the incorporation of sulfur content in amino acids in animal tissues [44]. Isotopic values of 0 to 5‰ have been recorded in reduced sulfur in hydrothermal systems (Fry et al, 1983), while values of 20‰ or more depleted have been measured in biologically produced H_2_S (Sakai *et al*., 1987). Considering the δ^34^S values reported in the sulfides of other hydrothermal vents [45], we assumed that organisms with positive values are either feeding on free-living bacteria or host symbiont bacteria that uptake reduced sulfur from the vents fluids. These organisms include Actiniaria sp. 1, Actiniaria sp. 2, Actiniaria sp. 5, *N*. cf *sandersi*, *O*. cf *akessoni*, and *P*. *laevis*. The low δ^13^C of the three actiniarid anemones, their high δ^15^N and their δ^34^S values close to those of the sulfides supports their potential symbiotic relationship with sulfide-oxidizing bacteria.

The ample difference in δ^34^S between the sulfates of the seawater and the sulfides from hydrothermal fluids results in organic matter of photosynthetic (~16‰ to 19‰) and chemosynthetic origin (-9‰ to 10‰) having distinctive δ^34^S values [46]. Most of the δ^34^S values of the fauna analyzed were negative or close to 0‰, deviating from the heavy values (+15 to +20‰) typically observed in marine animals supported by a photosynthetic production [47]. Similar low δ^34^S values (-27.7 to +5‰) were reported in the vent fauna from the Ogasawara Arc and Mid-Okinawa Trough [43] and Manus Basin vents [17].

All the δ^34^S values here reported facilitated the discrimination of PB from PTF vent faunas. PTF species exhibited the lowest, < -26.4‰ (Fig 3B), whereas PB species presented values higher than -14.55 ± 1.73‰, closer to the reduced sulfur values (0 ± 5‰) found in hydrothermal vent fluids [45]. The low values recorded in PTF fauna (< -20‰) corresponded to biogenic H_2_S [45]. *C*. *costaricana* presented the lowest δ^34^S values. These ratios were consistent with the assimilation of sulfur through a reservoir of sulfates impoverished by methanotrophic bacteria [31] that likely exist in PTF. Consequently, the organic sulfur contained in these vesicomyids is not of magmatic origin and must be attributed to H_2_S produced by the bacterial reduction of dissolved sulfates in the sediments/water interface. Such reduction can produce H_2_S with values between -20 and -70‰ [43].

The δ^34^S values proved to be useful in excluding methanotrophic species. The low δ^13^C value of *C*. *costaricana* (~-40.8) seemed to indicate that this vesicomyid hosts methanotrophic endosymbionts. However, methanotrophic species are expected to have high δ^34^S values around 21‰ [48]. *C*. *costaricana* presented lower δ^34^S values (-32.7‰), suggesting that it is a thiotrophic species rather than methanotrophic. The complementary analysis of both δ^34^S and δ^13^C contributed to elucidate the identity of species with this nutritional strategy. As referred earlier, no methanotrophic endosymbionts have been detected in its tissues.

The siboglinid worms *L*. *barhami* and *E*. *spicata* from PFT displayed similar low δ^34^S values, suggesting the exploitation of the same sulfur source. Such low values coincided with previous reports of sulfides supply through roots or opisthosoma [49].

The Actiniaria sp. 3 displayed the highest δ^34^S value (~15‰), indicating assimilation of mixed epipelagic photosynthetic and chemosynthetic products (range of 10‰ to 16‰) [14]. Van Audenhaege *et al*. [17] found δ^34^S values of 11 to 17‰ in the sponge *Abyssocladia dominalba*, which is consistent with an allochthonous source of sulfur derived from seawater sulfate. These values correspond to non-vent organisms. The input of photosynthetically derived POC or DOC is an additional source of nutrition to vent macroconsumers, although it is deemed negligible [14]. Presumably, 5–10% of the organic matter produced in the photic layer reaches a depth of 2,000 to 3,000 m [50]. When δ^13^C and δ^15^N values alone could not discriminate between inputs of photosynthetic and chemosynthetic material, very low δ^34^S values (relative to bottom water sulfate ~20.3‰) may serve to recognize the assimilation of photosynthetic organic carbon in the nutrition of the species [31]. The individual analysis of δ^15^N signatures allows distinguishing allochthonous carbon sources, but the interpretation could be confused because an enriched δ^15^N value can be attributed to the presence of more trophic levels in a site. However, the dual analysis of δ^34^S and δ^15^N values provides an unambiguous tool to detect a photosynthetic food source.

In this research, four sulfur sources used by the vent-fauna were identified: the reduced sulfur contained in the vent fluids, microbial sulfide oxidation (by free-living or symbiotic bacteria), the sulfides biologically produced by the microbial sulfate reduction, and the photosynthetic inputs. Of these, the microbial sulfide oxidation is the main contributor of sulfur to the macrofauna. Presumably, the bacterial sulfate reduction that produces depleted sulfides (≤20‰) only occurs in the Pescadero Transform Fault, since the most depleted values were recorded in the vent-fauna from this locality.

## Conclusions

Despite the proximity of the vent systems of Pescadero Basin and Pescadero Transform Fault, their physical, chemical, and geological settings differ considerably, which is reflected in their species diversity, trophic structure and the isotopic composition of the macrofaunal assemblages. δ^13^C allowed the identification of carbon sources derived from two principal carbon fixation pathways: the CBB, and the rTCA cycles. These cycles function more efficiently under optimum temperatures, CBB (<20°C) and rTCA (between 20°C and 90°C). Therefore, the temperature seems to be a driver of the predominance of CBB sources, or mixed carbon sources, in low-temperature vents of PTF. While both CBB and rTCA sources were similarly detected in PB, where a more extensive range of temperatures can be found. The species diversity was different among localities, displaying a short trophic web in PTF and a more complex food-web in PB, encompassing five trophic guilds as confirmed by the dual δ^13^C/δ^15^N approach. δ^15^N values fall among the typically depleted values recorded in vent-fauna. This indicates the assimilation of local nitrogen sources, predominantly NH_4_. The analysis of the δ^34^S ratios facilitated the discrimination of the fauna of PB from that of PTF, due to the predominance of biogenic sulfides in the latter locality in which the lowest values were recorded. Similarly, δ^34^S ratios allowed to exclude methanotrophic organisms and at the same time, to detect photosynthetic input in non-vent fauna, which has the capacity of assimilating chemosynthetic organic matter from venting site. This research represents a baseline of the trophic structure and interactions of the macrofauna of CP and PTF, although some links of the food web remain to be analyzed, such as the carbon, nitrogen, and sulfur sources, and the primary producers. The potential relationship between anemones and sulfur-oxidizing bacteria remains to be unraveled.
